# Lifehistory Trade-Offs Influence Women’s Reproductive Strategies

**DOI:** 10.1007/s40750-024-00236-3

**Published:** 2024-03-26

**Authors:** R. I.M. Dunbar, Sara Grainger

**Affiliations:** 1 Department of Experimental Psychology, University of Oxford, South Parks Rd, Oxford, OX1 3UD UK; 2 School of Biological Sciences, University of Liverpool, Liverpool, L69 3BX UK

**Keywords:** Lifehistory, Reproductive decisions, Socioeconomic class, Women, Contingent decisions

## Abstract

**Objective:**

In a UK national census sample, women from the upper and lower socioeconomic (SES) classes achieve parity in completed family size, despite marked differences in both birth rates and offspring survival rates. We test the hypothesis that women adopt reproductive strategies that manipulate age at first reproduction to achieve this.

**Methods:**

We use a Monte-Carlo modeling approach parameterized with current UK lifehistory data to simulate the reproductive lifehistories of 64,000 individuals from different SES classes, with parameter values at each successive time step drawn from a statistical distribution defined by the census data.

**Results:**

We show that, if they are to achieve parity with women in the higher socioeconomic classes, women in lower socioeconomic classes must begin reproducing 5.65 years earlier on average than women in the higher SES classes in order to offset the higher class-specific mortality and infertility rates that they experience. The model predicts very closely the observed differences in age at first reproduction in the census data.

**Conclusions:**

Opting to delay reproduction in order to purse an education-based professional career may be a high risk strategy that many lower SES women are unwilling and unable to pursue. As a result, reproducing as early as possible may be the best strategy available to them.

## Introduction

Evolutionary theory predicts that the expected duration of the reproductive life span should influence age at first reproduction: individuals who expect to die early should begin to reproduce earlier (Charnov, 1991; Stearns, 1992). There is some empirical evidence to support this prediction in humans. Low et al. (2008) showed that, across countries, female life expectancy is associated with age at first birth, with earlier onset of reproduction where mortality rates are high (see also Walker et al., 2006). Similarly, Lycett and Dunbar (2000) found that expected future reproductive lifespan was a significant factor influencing single women’s decisions whether or not to opt for an abortion. Guegan et al. (2001) reported that disease burden (interpreted as an index of prevailing mortality risk) predicts total fertility rates across human societies, while Quinlan (2007) found that, in societies where death rates are high (due to disease, famine or warfare), weaning is earlier (and maternal investment in offspring is reduced) compared to societies where conditions are more favourable. In sum, poor quality environments where the future is unpredictable favour a ‘fast’ lifehistory strategy. This commonly involves a suite of behaviours that comprises an early switch from investing in growth to investing in reproduction, high fertility and low parental investment (Bielby et al., 2007; Nettle, 2010).

Essentially similar effects have been noted at a within-society level. In a study of US urban environments, reproduction was shifted earlier in neighbourhoods which had higher mortality and morbidity rates (Geronimus, 1996; Geronimus et al., 1999; Wilson & Daly, 1997). Similarly, Nettle and Cockerill (2010; see also Nettle, 2011) reported that, in the UK, women’s age at first reproduction was, on average, 8 years earlier in poorer neighbourhoods than in neighbouring richer ones. Indices of maternal investment (such as birthweight and the duration of breastfeeding) are well known to vary with socioeconomic status (Dubois & Girard, 2006; Kohlhuber et al., 2008; Mortensen et al., 2008; Nettle, 2010). Similarly, many studies have noted that women begin reproducing earlier, reproduce more frequently, and invest less in each offspring in neighbourhoods where social and economic deprivation resulted in a shortened expectation of life (Nettle, 2010, 2011; Brooks-Gunn et al., 1993; McCulloch, 2001; Smith & Elander, 2006).

These lifehistory consequences of reproduction may be exacerbated in knowledge-based economies such as those prevailing in most of the industrialised world. The need to ensure that offspring are competitive in terms of education, wealth and/or social/economic opportunities favours a reduction in fertility and a corresponding investment in offspring quality (Becker & Lewis, 1973; Rogers, 1990; Mace, 1998). In such contexts, future earning potential may be as important as longevity and may favour the postponement of reproduction in order to allow investment in social or career prospects that offer enhanced mate choice opportunities or the acquisition of resources that can be invested in offspring. In such contexts, women who can afford to do so (and hence, especially, women from higher socioeconomic [SES] classes) should be more willing to delay the onset of childbearing in order to further their educational and career opportunities.

Although many of these contingent effects are well known, they have typically not been examined together in a lifehistory context (Nettle, 2010, 2011; Liu et al., 2012). Here, we use a simulation model to investigate the lifetime fertility consequences of postponing reproduction in the interests of furthering career opportunities when these are likely to have significant socioeconomic consequences. We assume (following Mace, 1998) that, in a socio-economic environment where the costs of producing children who will be able to function effectively in the adult economy are high (due to the high costs of education and/or placing children in a socio-economically advantageous position), parents will reduce family size to that in which they can realistically afford to invest. All else equal, women from higher socioeconomic classes who benefit from higher fecundity and lower mortality ought to have higher lifetime fertility (completed family size – in effect, fitness). Data from Finnish historical demography records confirm that, if age at first reproduction is held constant, women from higher socio-economic classes out-reproduce less well-off women (Liu et al., 2012).

Nettle (2008) suggested that, under contemporary socioeconomic regimes, women from lower socioeconomic classes might be content to match the reproductive outputs of higher SES women rather than try to out-compete them, in part because of the high cost of competing in a knowledge-based economy. In addition, they are more likely to incur reduced paternal investment (of which father absence is a common, though not necessarily the only, component) and a higher risk of being a single-parent family, both of which exhibit a marked socioeconomic class gradient. This may add to the economic costs of rearing for mothers in lower SES classes, since the burden of rearing will fall disproportionately on their shoulders and may encourage them to favour a satisficing rather than a maximizing reproductive strategy. A satisficing strategy at least enables them to remain in the evolutionary game in a way that might allow their offspring to benefit from better conditions for reproduction in the future.

In order to offset all these costs, women in the lower socio-economic classes will need to begin reproduction earlier, and/or continue reproducing later, if they are to arrive at the same final family size as women in the higher social classes. We thus hypothesise that differences in age at first reproduction between socioeconomic classes may become part of a strategy aimed at achieving the same desired family size under different constraints. Psychologically, this might be interpreted as reflecting a view that competing in the education game is less profitable that commencing reproduction as early as possible. This does not have to be a consciously held view, but it may nonetheless be a subconsciously processed attitude. However, our concerns here are not with the motivations that individuals have but with the conditions under which parity of fertility becomes possible.

Parity in fertility could only be the case, however, if it is true that early reproduction does not increase the risks of unsuccessful reproductive events (e.g. higher spontaneous abortion or postnatal mortality rates) – i.e. that early reproduction does not incur significant costs in and of itself. This does not mean that there should be no costs to very early reproduction; rather, it means that there should be no additional costs to reproducing earlier than women from wealthier families, providing this does not occur too early. Data for first pregnancies from the British Cohort Study confirm that maternal age currently has no predictive power for a successful outcome for first pregnancy when controlling for social class (*N* = 9475: β=-0.001, Wald = 0.010, df = 1, *p* = 0.920). This is consistent with many studies showing that the negative effects of young maternal age have previously been exaggerated by a failure to consider the confounding effects of parity and social class (Arif et al., 1998; Malik et al., 1997; Reichman & Pagnini, 1997).

In order to test the prediction that the higher mortality and unsuccessful pregnancy rates experienced by women in the lower social classes would result in lower lifetime reproductive success compared to women in the higher social classes if they delayed age at first reproduction, we developed a simulation model using class-specific mortality and fertility data to calculate the probability of women surviving and reproducing in each year between the ages of 15 and 45 years. Our aim is to ask whether social class differences in age at first reproduction could be due mainly to class differences in experienced fertility and mortality, and individual women’s attempts to optimise completed family size (i.e. lifetime reproductive output) under different constraints. We test the model against the observed class differentials in age at first reproduction.

## Methods

We develop a Monte Carlo simulation of women’s reproductive life-histories for women who choose to commence reproduction in each of the odd-numbered years between ages 15 and 45, for each of four main socioeconomic classes (I + II, IIIN, IIIM and IV + V; recently relabelled as AB, C1, C2 and DE, respectively) as defined by the UK Registrar General. Class I/II (AB) is defined as higher and intermediate managerial, administrative and professional occupations (currently 23.3% of the adult population); class IIIN (C1) as supervisory, clerical, and junior managerial, administrative and professional occupations (32.8% of adult population); class IIIM (C2) as skilled manual occupations (21.3% of population); and class IV/V (DE) as semi-skilled and unskilled manual occupations, unemployed and lowest grade occupations (22.6% of population) (ONS, 2024).

We ran 1000 simulations for each cohort (a total of 64,000 simulated individual lifehistories in all). The equations that define the event probabilities are given in Table 1. These equations were directly obtained from contemporary UK national statistics databases (ONS, 2001). The Health Survey for England and the British Cohort Study were supplied by the UK Data Archives held at the University of Essex. Mortality Statistics for the UK 1998 and the Health Inequalities: Supplementary Dataset 1987–1991 were supplied by the Office of National Statistics.

**Table 1:** Parameter values used in the model

Probability of:	Social class I&II	Social class IIIN	Social class IIIM	Social class IV&V
Probability of infertility ^i^	0.1466	0.1691	0.1705	0.1806
Probability of conceiving within year (women aged ≤ 25) ^ii^	0.6728	0.6683		0.6658
Probability of conceiving within year (women aged 26–30) ^ii^	0.6827	0.6778		0.6753
Probability of conceiving within year (women aged 31–35) ^ii^	0.5628	0.5587		0.5567
Probability of conceiving within year (women aged ≥ 36) ^ii^	0.4982	0.4946		0.4928
Probability of conception leading to birth by x = pregnancy number ^iii^	-0.0080 × ^2^ + 0.0892x + 0.0369 (r^2^ = 0.93)	-0.0112 × ^2^ + 0.0821x + 0.0607 (r^2^ = 0.85)	-0.0032 × ^2^ + 0.0410x + 0.0683 (r^2^ = 0.89)	-0.0057 × ^2^ + 0.0502x + 0.0455 (r^2^ = 0.72)
Probability of conception leading to birth by x = interbirth interval (years) ^iii^	0.01940x + 0.779 (r^2^ = 0.97)			
Probability of offspring dying by age 1 year ^iv^	0.0119	0.0139	0.0143	0.018
Probability of offspring dying by age 24 years ^v^	0.0094	0.0092	0.0125	0.010
Probability of mother dying within year, as a function of her age (x = age, in years) ^v^	0.0000058 × ^3^ -0.0006 × ^2^ + 0.0152x-0.0711 (r^2^ = 0.93)	0.0000057 × ^3^ – 0.0006 × ^2^ + 0.0148x-0.0697 (r^2^ = 0.93)	0.0000054 × ^3^ – 0.0005 × ^2^ +0.0132x-0.0602 (r^2^ = 0.95)	0.0000053 × ^3^– 0.0005 × ^2^ + 0.0131x-0.0609 (r^2^ = 0.95)

^i^ Based on a population infertility rate of 16.67% weighted according to the effect of smoking^10^ and the proportion of people who smoke in the given social class

^ii^ Based on the probability of conception according to age ^17^ weighted according to the effect of smoking^10^ and the proportion of people who smoke in the given social class

^iii^ Data from British Cohort Study

^iv^ Mortality statistics for the UK 1998 (ONS)

^v^ Health Inequalities: Supplementary Dataset 1987–1991

**Fig. 1:**
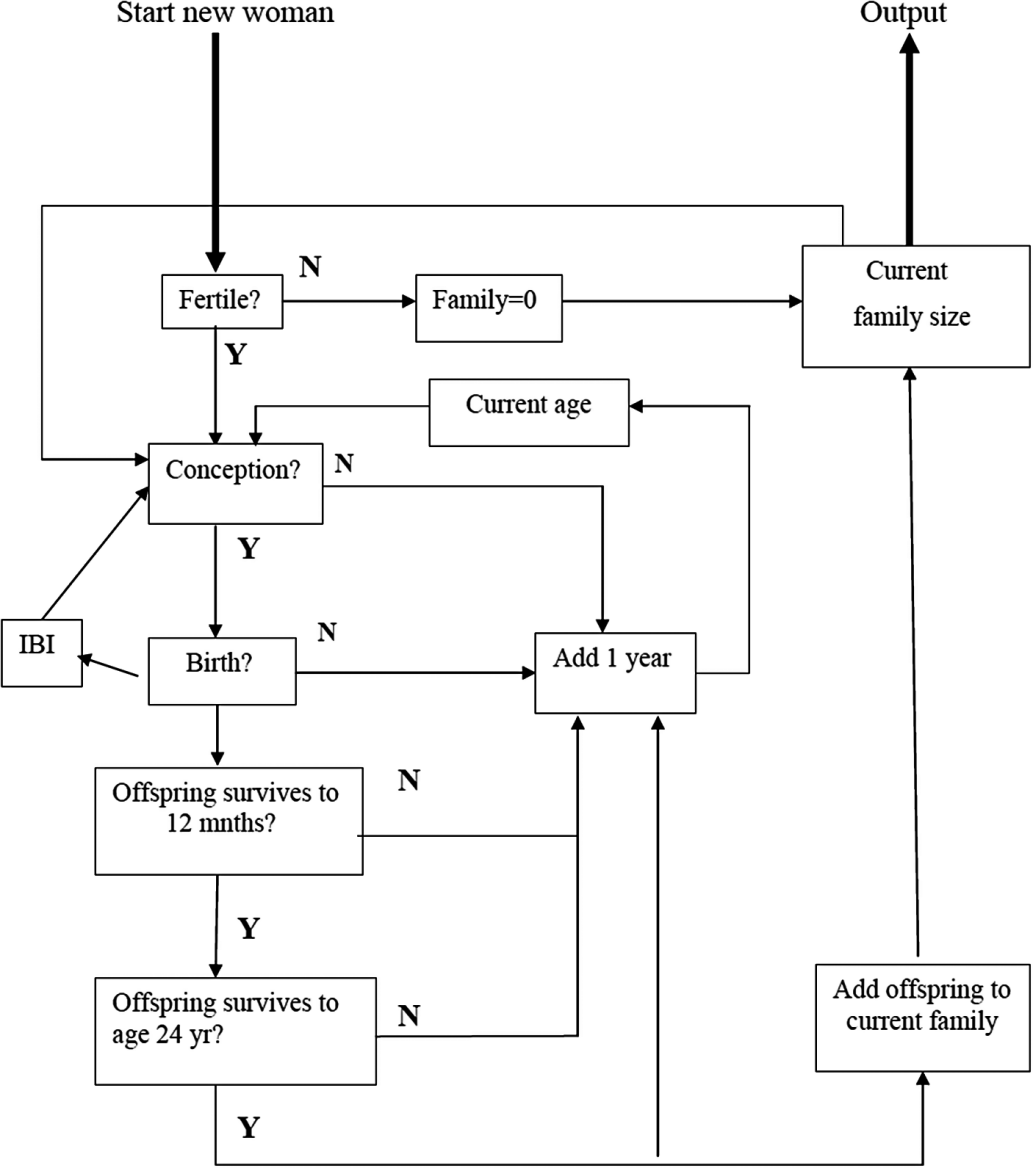
Flow chart for lifetime reproductive output simulation model. The model calculates an individual female’s net lifetime reproductive output as a function of her socioeconomic class, taking into account the risks of class-specific mortality for both the mother and each successive offspring conceived. Offspring survival is calculated to age 24 years. Fecundity, spontaneous abortion and mortality rates are based on national rates for England and Wales for the decade ending in 1991 given by the UK’s Office of National Statistics (ONS). For simplicity, all women are assumed to achieve menarche at age 15 years and menopause at age 45 years

The flow chart for the model is shown in Fig. 1. For each reproductive history, the class-specific probability of infertility is first used to determine whether or not the woman is infertile (i.e. never reproduces, irrespective of whether the cause is organic infertility or never marries and has no illegitimate children), with completed family size being 0 if she is. For fertile women, a random number generator was used in conjunction with the probability density functions provided by the class-, age- and birth-order-specific probabilities to determine whether or not (a) the woman conceives that year, (b) conception leads to birth (given natural abortion rates only), (c) the infant survives to become a (potentially) reproductive adult (taken to be age 24 years) and (d) the mother survives to the next age interval, for each successive year from the selected age at first reproduction. This fertility cycle is then repeated for the successive age classes until the woman dies or reaches menopause after age 45.

Completed family size was taken to be the total number of children that survived to age 24 years accumulated over the woman’s entire reproductive history from age 15 years to either age at death or the onset of menopause (at age 45 years), whichever is the earlier. We do not explicitly include marital status (i.e. the risk of single parenthood) in the model; its effects are, however, automatically included in the class-specific equations for birth rates and maternal and offspring mortality. Our concerns here are with the outcome measures, not their causes. The aim of the model is to identify the boundary conditions under which parity of reproductive output occurs.

## Results

**Fig. 2:**
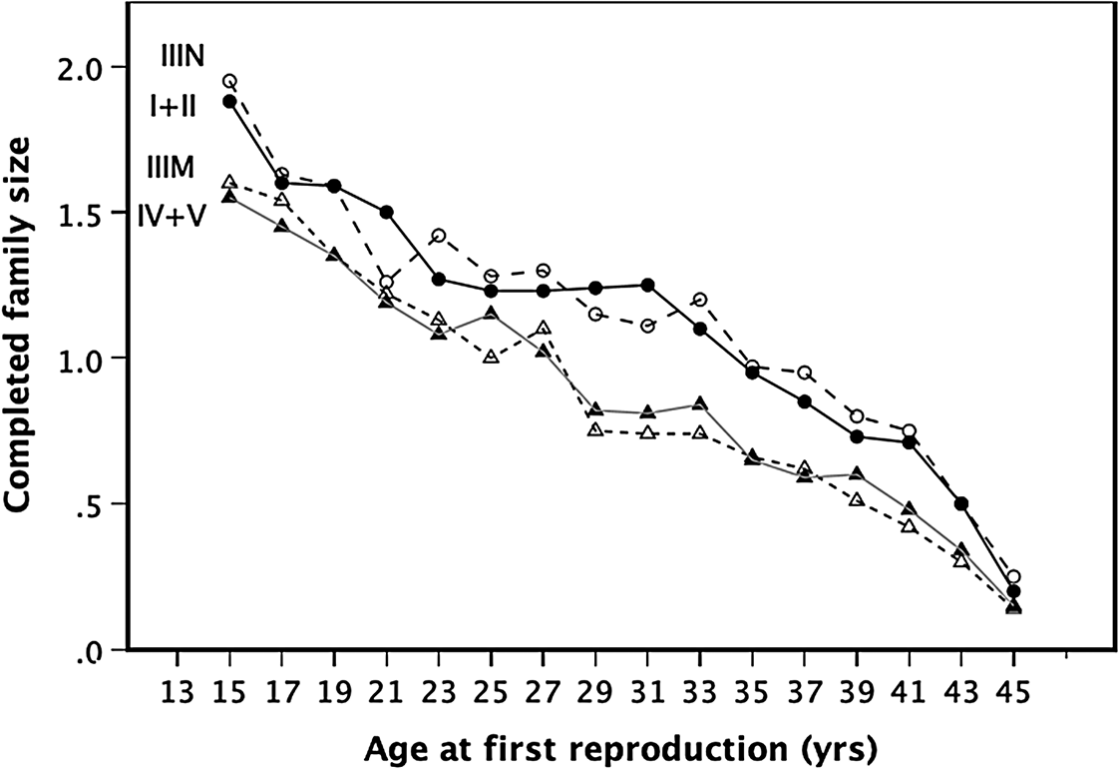
Mean completed family size predicted by the simulation model for women from different social classes who start reproduction at different ages. For each age at first reproduction and each social class, the reproductive lifehistories of 1000 women were simulated using a model parameterized with the equations given in Table 1

Figure 2 plots the net completed family size (lifetime reproductive output) for women in the four SES classes as a function of age at first reproduction. These show that the different rates of mortality, subfertility, and unsuccessful pregnancy between the social classes are great enough to result in significantly different patterns of lifetime reproductive success between women in different social classes who begin reproduction at the same age. There were significant effects of both age at first reproduction and social class on lifetime reproductive output (F_3,3996_=92.72, *p* < 0.001; age F_1,3996_=247.75, *p* < 0.001, class F_1,3996_=30.33, *p* < 0.001). Thus, delaying reproduction reduces reproductive output for women in every social class but, crucially, *within age categorie*s completed surviving family size is always lower for the women in social class IV/V than it is for women in social classes I/II. These within-cohort effects can be attributed to the cumulative costs in terms of fecundity and survival that characterise women of different SES classes (see Table 1). Though, individually, these costs may seem small, between them they add up to effects of significant magnitude.

**Fig. 3:**
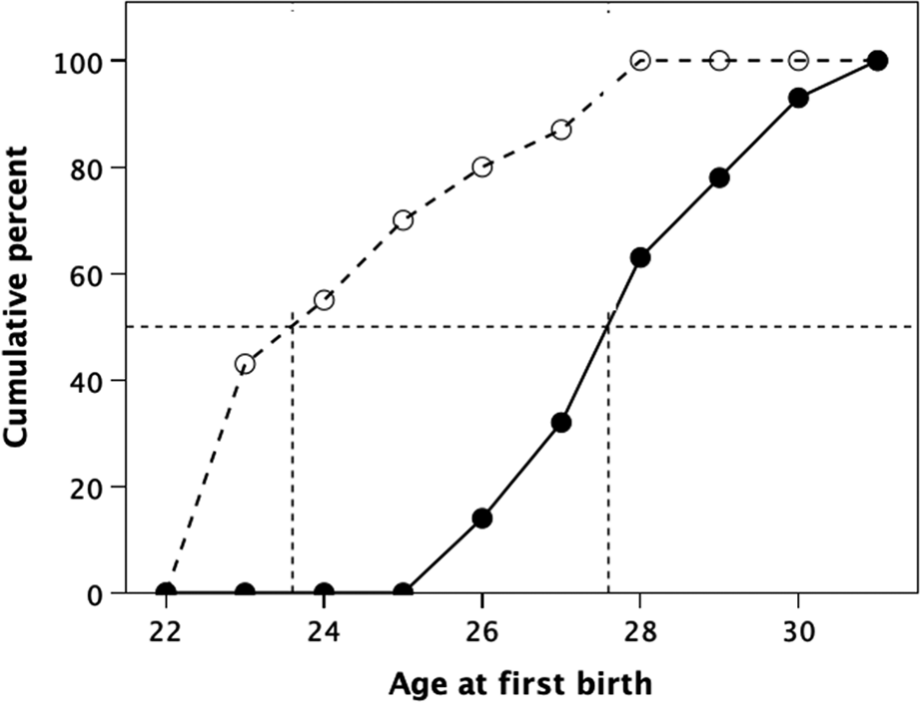
Cumulative percentage of actual age at first birth for women of social (SES) classes I/II and IV/V in England and Wales in 1970–2000. Mean age at first birth is indicated by the dotted lines down from the 50th centile line: 23.6 years for class IV/V versus 27.6 years for class I/II. Source: ONS (2001)

Across the full range of age classes represented in Fig. 1, the mean age difference at which women in social class IV/V achieve net reproductive parity with women in social class I/II is 5.65 years (averaged across 15 age classes). This corresponds well to the observed pattern: in England and Wales, the mean age at first reproduction between 1970 and 2000 was 27.6 years in social classes I & II and 23.6 years in social classes IV & V (Fig. 3). The mean difference between the two classes (4.6 years) is highly significant (comparison of annual means against a null hypothesis of Δµ = 0: *F*_1,60_=74.8, *p* < 0.001). The difference in mean age at first reproduction between the classes predicted by the model is not significantly different from the observed value (*z* = 1.270, *p* = 0.204). In other words, women in social class IV/V have to start reproduction up to half a decade earlier than their counterparts in social class I/II if they are to gain parity with them in completed family size. More importantly, in order to out-compete women in the higher social classes, these women would either need to begin reproduction earlier still (i.e. as late teenagers: Fig. 3) or continue reproducing much later. They do not, in fact, appear to do either.

## Discussion

Our analysis starts from the empirical observation that, at least in the UK, the upper and lower socioeconomic classes are at reproductive parity: on average, they have very similar completed family sizes (i.e. number of offspring that survive to an age where they can themselves start to contribute to the parents’ fitness by producing grandchildren). Yet they differ in rates of infertility, reproductive failure and infant and maternal mortality risk (Table 1). We show that, to achieve parity with their better-off sisters, women in lower socio-economic classes would need to begin reproducing about half a decade earlier than women in the higher socio-economic classes. Women in this sample do indeed differ in the onset of reproduction by exactly the amount required to match the lifetime reproductive outputs.

The conventional view in demography and social policy has tended to be that early reproduction and pronatalist attitudes are largely a consequence of differences in education or attempts to access social resources (e.g. housing) (Peckham, 1993; Cleland, 1995; Franklin & Corcoran, 2000; Geronimus, 2003; Duncan, 2007). However, while education and educational opportunities may be precipitating factors, our results point to the importance of a strategic lifehistory perspective in understanding human reproductive behavior. They suggest that the higher birth rates and earlier onset of reproduction characteristic of some women may not simply be a consequence of pronatalist attitudes. Instead, they might actually reflect a sensitive strategic response to class-specific risks of reproductive failure in a context where the great majority of women of all classes in fact aim at much the same target completed family size. In other words, early reproduction may not be a mistake as such, but an adaptive strategy (Nettle, 2010). Indeed, Nettle (2010) offers a compelling argument for the causal logic being that economic circumstances affect lifehistory traits and these in turn affect age at first reproduction rather than the other way around (as has been commonly assumed).

Since the decision about when to start reproduction is made well in advance of any decision about when to cease reproduction, we interpret it as mainly reflecting decisions that women make about whether they can afford to delay the onset of reproduction in order to invest in careers in the light of the effect that career-dependent movement from one social class to another may have on the lifehistory parameters with which they will subsequently have to cope once they start reproducing. In effect, women with limited expectations of future career opportunities (mainly those in lower SES classes whose educational opportunities are limited) should prefer to opt out of career-based life trajectories in favour of early reproduction. Doing so provides them with significantly higher fitness than the opportunities offered by trying to compete in an economic market for which they may be socially or educationally ill-equipped.

Our concern here has been with establishing the boundary conditions under which parity of fertility can be achieved. We have not been concerned to establish what factors influence these individual processes. Rather, our concern has focussed on what options the women have, given the circumstances under which they have to make their decisions. In other words, we have been interested only in the outcome of each process, not their causes. For the sake of completeness, however, we briefly consider some aspects of the causal processes underlying these effects that might be implicated in our results.

The decision to begin reproduction earlier may well depend on whether a woman is likely to die before menopause (i.e. the end of the reproductive period). In the past, differential mortality was considerable since death rates were high in low SES women. In recent decades, however, the differential has narrowed, with mean age at death for women now exceeding 70 years in both deprived and wealthy communities (Rashid et al. 2021). Since this is well beyond the age of menopause, it cannot influence fertility as such (though it might have implications for grandparental investment).

A variety of variables are known to influence both age at menarche and patterns of sexual activity (Thomas et al., 2001; Romans et al., 2003). Father absence is one well known factor that has been studied in some considerable depth (Draper & Harpending, 1982; McLanahan & Teitler, 1998; Ellis et al., 2003; Grainger, 2004; Guo et al., 2020; DelPriore et al., 2021; Hehman & Salmon, 2021). Belski et al. (2012) found, in a US sample, that degree of maternal harshness when the child was aged 54 months correlated strongly with an earlier age at menarche and greater sexual risk-taking during teenage years in daughters. Though they did not consider parental socioeconomic status, it is plausible to suggest that this is likely to be at least partially correlated with rearing behaviour (Conger & Donnellan, 2007). In the present study, however, early menarche is unlikely to play a significant role because the mean age at first reproduction is well beyond menarche, even in the lowest SES class women (Fig. 3). Conversely, single parenthood is stressful (Flouri et al., 2016) and premature maternal death necessarily impacts on the survival of younger children, and both may favour premature termination of reproduction. Higher mortality rates in teenage and older mothers (see Table 1), especially in the economically less well off classes, may introduce stabilising selection.

A number of studies have linked late onset of reproduction and delayed marriage to increasing difficulty in finding suitable partners (assortative mating) once women embark on career-oriented life strategies, and in particular the effect that the prolonged education necessary for professional careers has on the time at which women finally enter the workplace (Becker, 1981; Oppenheimer, 1988). In an analysis of the UK 1958 cohort longitudinal sample, for example, Nettle and Pollet (2008) found that the proportion of childlessness increased with income in women (but decreased in men). One caveat, however, may be that high rates of childlessness may not necessarily translate into low mean fertility. Lawson and Mace (2011) cautioned that there may be benefits to having fewer children for wealthier families in terms of parental investment in future social and economic opportunities. One of those benefits may be reduced childhood morbidity and mortality, and hence greater certainty in successfully rearing all offspring that are born.

Whereas delayed marriage (and hence delayed reproduction) has often been viewed as an unfortunate by-product of education and career opportunities (with potentially adverse consequences at both individual and societal levels), there is likely to be a strategic component: all such decisions are necessarily contingent on current costs and benefits. In evolutionary terms, individuals *should* take note of their circumstances and opportunities in choosing when and how often to reproduce. That these decisions are contingent on circumstances was noted by Lycett and Dunbar (2000) in their analysis of UK abortion rates. In many such cases, the choice is between a low variance, low risk strategy and a high variance, high risk strategy.

Contrasts of this kind are common in real-life decision contexts. Examples include the difference between peace chiefs and war chiefs among the Cheyenne and other Plains Indians: peace chiefs (who inherited their titles and avoided conflicts) lived long lives and married early whereas war chiefs (most of whom were orphans with few prospects) led war bands during inter-tribal conflicts, incurred high risks of being killed and married late, but typically had higher fertility when they did marry (Dunbar, 1991). Those who can afford to take the risk should opt for the reproductively high risk strategy, while those who cannot should opt for the safer low risk strategy. In an evolutionarily stable strategy set, the mean fitness payoffs should be equal when the frequencies are equal – as, in fact, they are in the present case (because the SES classes are defined to be approximately equal in size: see *Methods*).

In sum, our results suggest that women’s reproductive strategies may be more subtle and have a much longer time perspective than is often assumed. In the present case, the results appear to constitute a form of fitness-matching (i.e. women try to match a culturally “agreed” optimal family size produced by those in the better-off social groups) in order to avoid being disadvantaged in fitness terms. It is possible that this reflects a “best of a bad job” solution, but this could only be established from interview data and is beyond the scope of the present study.

## Data Availability

The data are available in the published sources referenced in the Methods. The program for the model is available on request from RD.
